# Fabrication of Poly(**ε**-caprolactone) Scaffolds Reinforced with Cellulose Nanofibers, with and without the Addition of Hydroxyapatite Nanoparticles

**DOI:** 10.1155/2016/1596157

**Published:** 2016-10-31

**Authors:** Pedro Morouço, Sara Biscaia, Tânia Viana, Margarida Franco, Cândida Malça, Artur Mateus, Carla Moura, Frederico C. Ferreira, Geoffrey Mitchell, Nuno M. Alves

**Affiliations:** ^1^Centre for Rapid and Sustainable Product Development, Polytechnic Institute of Leiria, 2430-028 Marinha Grande, Portugal; ^2^Coimbra Institute of Engineering, Polytechnic Institute of Coimbra, 3030-199 Coimbra, Portugal; ^3^Institute for Bioengineering and Biosciences and Department of Bioengineering, Instituto Superior Técnico, Universidade de Lisboa, 1049-001 Lisboa, Portugal

## Abstract

Biomaterial properties and controlled architecture of scaffolds are essential features to provide an adequate biological and mechanical support for tissue regeneration, mimicking the ingrowth tissues. In this study, a bioextrusion system was used to produce 3D biodegradable scaffolds with controlled architecture, comprising three types of constructs: (i) poly(*ε*-caprolactone) (PCL) matrix as reference; (ii) PCL-based matrix reinforced with cellulose nanofibers (CNF); and (iii) PCL-based matrix reinforced with CNF and hydroxyapatite nanoparticles (HANP). The effect of the addition and/or combination of CNF and HANP into the polymeric matrix of PCL was investigated, with the effects of the biomaterial composition on the constructs (morphological, thermal, and mechanical performances) being analysed. Scaffolds were produced using a single lay-down pattern of 0/90°, with the same processing parameters among all constructs being assured. The performed morphological analyses showed a satisfactory distribution of CNF within the polymer matrix and high reliability was obtained among the produced scaffolds. Significant effects on surface wettability and thermal properties were observed, among scaffolds. Regarding the mechanical properties, higher scaffold stiffness in the reinforced scaffolds was obtained. Results from the cytotoxicity assay suggest that all the composite scaffolds presented good biocompatibility. The results of this first study on cellulose and hydroxyapatite reinforced constructs with controlled architecture clearly demonstrate the potential of these 3D composite constructs for cell cultivation with enhanced mechanical properties.

## 1. Introduction

Tissue engineering (TE) approaches have a high potential for the development of new therapeutic strategies for medical applications, which have led to an increasing number of research and development studies by academic and industry communities. In recent years, the utility of cellulose fibres in biomedical applications has gained distinctive attention by the scientific community, due to the unique combination of its properties such as nontoxicity, biocompatibility, biodegradability, low cost, and high mechanical modulus [1, 2]. This natural polymer is a homopolysaccharide formed by linearly connected D-glucose units condensed through the *β* (1–4) glycosidic bonds [3, 4]. It is the most available polymer in nature and is widely available from several sources. Furthermore, it can be synthetized by woody plants, several kinds of algae and fungi, grasses, and some species of bacteria [5–7]. The applications of cellulose include coatings, filtration, catalysis, sensors, and medicine [6, 8]. Additionally, its use is particularly interesting for biomedical applications because nanofibers have been successfully used as highly effective reinforced fillers for numerous different polymers, enhancing the mechanical properties of the composites and improving cell biocompatibility [9–11]. Lastly, the interaction of polymer blends has been of intensive interest due to the number of valuable properties and strong economic incentives.

On the other hand, porous composite scaffolds have been extensively used in TE approaches, as a support for cell attachment, cell growth, and tissue regeneration [12]. An ideal scaffold must be able to provide the essential properties and function to satisfy simultaneously the biological and mechanical requirements for optimal tissue regeneration [13]. To reach these requirements, several studies have been developed based on (i) 3D porous scaffolds with arbitrary architecture (uncontrolled pore size and spatial distribution); (ii) 3D porous scaffolds with hybrid architecture (pore size and spatial distribution partially controlled); and (iii) 3D porous scaffolds with controlled architecture (pore size and spatial distribution) [14]. Each of these approaches have advantages and drawbacks; the fact that having a controlled architecture may bridge the gap between produced scaffolds and native tissue is accepted by the scientific community.

Despite the progress achieved towards the development of structures as biological substitutes, the development of 3D biodegradable scaffolds with improved mechanical and biological properties remains a goal to be achieved. The architecture and mechanical properties of such scaffolds are important to promote further cellular activities and neotissue development. The properties of the scaffolds previously developed aiming at bone regeneration are reviewed elsewhere, with porosities varying widely from 20 to 90% [15]. Importantly, not only a reasonable high porosity, but high pore connectivity and surface area are essential to promote an initial efficient scaffold seeding by cells and metabolite transport and in further states efficient scaffold colonization with formation of continuous tissue across the full scaffolds 3D structure. For bone applications, Rouwkema et al. [16] had pointed out a minimal size of 100 *μ*m to allow oxygen and nutrients diffusion. Likewise, an optimal size of 200–350 *μ*m has been recommended [17] to facilitate both cell attachment and proliferation, for efficient tissue ingrowth.

Poly(*ε*-caprolactone) (PCL) is one of the most common medical approved linear aliphatic polyesters [18]. It is a hydrophobic and semicrystalline polymer, for which its crystallinity tends to decrease with a molecular weight increase [19]. Furthermore, its good solubility, low melting point (59–64°C), and exceptional blend-compatibility have motivated extensive research over its potential applications in the biomedical field [20]. As cellulose nanofibers (CNF) and PCL are biodegradable and semicrystalline polymers, there are several advantages to blend these two polymers. However, the dispersion of hydrophilic CNF in a hydrophobic thermoplastic to obtain a homogeneous composite is a paramount challenge, demanding more research on methods able to achieve it through the improvement of their interfacial adhesion properties [21]. For the preparation of polymer/cellulose nonhydrosoluble composites, conventional methods, such as solvent casting, usually require cellulose modified by surface coating or grafting to achieve good dispersion [1, 22].

For this research, semicrystalline PCL-based scaffolds with controlled architecture were produced. Aiming to examine the effects of cellulose in those constructs, they were reinforced with cellulose nanofibers with and without the addition of hydroxyapatite nanoparticles (HANP). Scaffolds were produced by extrusion, a layer-by-layer process, which results in a nanocomposite material that gathers unique proprieties: biodegradability resulting from PCL ester bonds breakdown, biocompatibility tailored for bone tissue formation through the use of hydroxyapatite, and mechanical properties provided by a nanostructure obtained with a combination of CNF and HANP. The nanocomposites were characterized by optical microscope, DSC, TGA, compression testing, and* in vitro* cytotoxic techniques. The present work provides a proposal to obtain biodegradable composites which can be further used in biomedical applications.

## 2. Materials and Methods

### 2.1. Materials

In this work PCL polymer (CAPA® 6500) from Perstorp Caprolactones (Cheshire, United Kingdom) with a molecular weight of 50 kDa was used. The CNF 3% (w/v) (Curran® Slurry) were provided by the Cellucomp (Burntisland, United Kingdom) and the HANP (≥97%, synthetic) with a particle size less than 200 nm was obtained from Sigma-Aldrich (Saint Louis, USA). Nanocomposites were produced using N,N-Dimethylformamide (DMF) from Merck KGaA® (Germany).

### 2.2. Composites Preparation

PCL pellets were dissolved in DMF at 50°C. The solution was deposited in Petri dishes and dried at controlled environment on an orbital shaker (KS 4000 i control, IKA, Germany) at 25°C for 48 hours. The PCL/CNF composite was prepared by solvent casting using lyophilized CNF. Cellulose aqueous samples were frozen at −40°C and then freeze-dried under vacuum (2 × 10^−3^ mbar with a ILMVAC GmbH vacuum pump) at −45°C using a FreeZone 4.5 freeze-drying equipment (from LABCONCO Corporation, Kansas, USA) for 72 hours. The frozen water was removed from the cellulose samples, initially by sublimation (primary drying) and then by desorption (secondary drying).

The corresponding membranes were prepared through the dissolution of PCL pellets (99% (w/w)) and CNF 1% (w/w) in DMF at 50°C, separately. CNF solution preparation includes sonication of the CNF at 100 W for 10 min, using an ultrasonic homogenizer (UP200Ht, Hielscher, Ultrasound Technology). After obtaining two homogeneous solutions, they were mixed using a magnetic stirrer (500 rpm) for 10 min. The PCL/CNF solution was deposited in Petri dishes and dried using the same methodology used for the production of PCL membranes.

The membranes of PCL/CNF/HANP were produced keeping the concentration of CNF at 1% (w/w) and adding 5% (w/w) of HANP in DMF. After complete dissolution, the obtained solution was deposited in Petri dishes and dried in a controlled environment, similar to PCL and PCL/CNF membranes.

### 2.3. 3D Scaffolds Production

**Figure 1:**
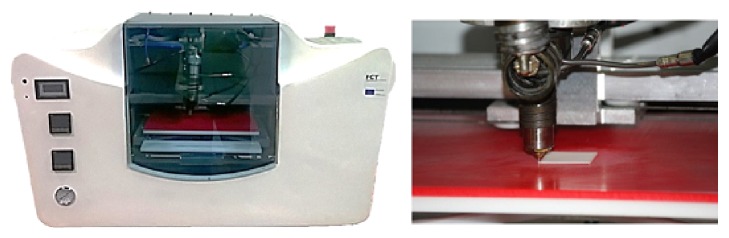
Bioextruder system, developed by the Centre for Rapid and Sustainable Product Development, Polytechnic Institute of Leiria.

The obtained membranes were processed by extrusion using a Bioextruder® system (Figure 1), developed by the Centre for Rapid and Sustainable Product Development, Polytechnic Institute of Leiria [23]. The 3D scaffolds were produced by fibre deposition with 300 *μ*m diameter, 350 *μ*m pore size, and 0°/90° lay-down pattern. These parameters took in consideration that a minimum pore size of 100 *μ*m is required for the diffusion of nutrients and oxygen for cell survival and proliferation. Furthermore, it has been shown that pore sizes up to 350 *μ*m are optimal for bone tissue ingrowth [24]. The process conditions used for all scaffolds were 20 mm/s of deposition velocity, 50 rpm of screw rotation velocity, and 90°C of liquefier temperature.

### 2.4. Morphological Analysis

The surface morphology of all produced membranes was examined by optical microscopy (Daffodil MCX100, Micros Austria) at a magnification of 40x. Additionally, micro-computed tomography (Micro-CT) scans of the scaffolds were performed using a SkyScan microtomograph model 1174 by Brucker Company (Brussels, Belgium). The CT system was operated with a rotation step of 0.7 degrees, voltage of 50 kV, exposure time of 2300 ms, and a current of 800 *μ*A with a nominal resolution of 16.65 *μ*m/pixel. The micro-CT analyses allowed the visualization of the internal and external morphologies of the samples as well as the calculation of porosities. The reconstructed set of slices was viewed in SkyScan Data Viewer program, 3D realistic images were made with CTvox software, and porosity values were calculated through CTan software.

### 2.5. Contact Angle Measurement

The wettability of the scaffolds was evaluated by static contact angle measurement at 10s on a Theta Lite optical tensiometer (Attension, Finland). A water droplet was poured on the surface of solid samples and the contact angle was measured by OneAttension 1.0. software (Attension). Aiming to increase its reliability, 15 measurements were performed for each scaffold type.

### 2.6. Thermal Analysis

A STA 6000 (Perkin Elmer®) was used for thermal analysis of the materials. Samples of 6 mg were placed in alumina pans and empty pans were used as reference. All samples were first heated at a range of 30–120°C at a heating rate of 10°C/min and held isothermally for 10 min to mitigate any prior thermal history. Afterwards, the samples were cooled to 30°C at 10°C/min and then reheated to 120°C at the same rate. After each test, the melting point region from the thermograph was analysed to determine the heat of fusion (Δ*H*_*m*_) and the melting temperature (*T*_*m*_); the crystallization region was analysed to determine the crystallization temperature (*T*_*c*_) of all samples. To evaluate the thermal degradation of the materials, the samples were exposed to a temperature ramp from 30°C to 600°C, at a heating rate of 10°C/min. The flow rate of nitrogen was 20 mL/min during all the runs.

### 2.7. Mechanical Analysis

Compression tests were performed to evaluate the effect of CNF and HANP addition on the mechanical properties of the PCL scaffold. The tests were conducted according to ASTM standards, using a ZWICK Z100, with a cross-head displacement speed of 1 mm/min. Mechanical testing was carried out using scaffolds samples in the dry state, with a length of 4 mm, a width of 4 mm, and a height of 8 mm. Stress-strain data were computed from load-displacement measurements and the compressive modulus (*E*) was determined from the elastic region of the obtained curves.

### 2.8. Cytotoxicity Assessment

*In vitro* cytotoxicity assessment was performed according to ISO standard 10993-5:2009, as described elsewhere [25]. Direct contact (qualitative) and extract (quantitative) assays were performed. Samples were sterilized in 70% ethanol and UV light overnight and then washed with phosphate buffered saline (PBS, Gibco®). Mouse fibroblasts L929 were cultured in Dulbecco's modified Eagle's medium (DMEM, Gibco), supplemented with 10% Fetal Bovine Serum (FBS, Life Technologies), and seeded on 24-well plates (1.5 × 10^5^ cells/well) to obtain approximately 80% of confluence, that is, well plate surface covered by adherent cells. The plates were incubated overnight (37°C; 21% O_2_; 5% CO_2_).

Scaffold samples were submersed in the medium and kept in the incubator for 72 hours to obtain the extracting culture media enriched with any eventual solutes that may had leach from the scaffolds. Then, for the extract assay, the culture medium of the cells was discarded and replaced with extracting culture media for further cultivation of the adherent cells for 24 hours. Cell activity of such cultures was assessed by a 3-(4,5-Dimethylthiazol-2-yl)-2,5-diphenyltetrazolium bromide (MTT, Sigma) protocol (extract assay). As controls, fresh medium was used as negative control and latex was used as positive control. All the conditions were tested in triplicate. Direct contact assays were carried out by placing the scaffolds on top of the cells layer at 80% confluence and incubated for another 24 h. After this, photographs were taken in the optical microscope for the direct qualitative contact assay.

### 2.9. Statistical Analysis

Normality and homoscedasticity assumptions were checked by Shapiro-Wilk and Levene tests, respectively. Descriptive statistics (mean and standard deviation) were calculated for all dependent variables. The significance of differences between types of scaffolds was evaluated by analysis of variance (one-way ANOVA, with Bonferroni* post hoc* test). All statistical procedures were performed using SPSS 23.0 (Chicago, IL, USA) and the G-Power 3.1.9.2 for Windows® (University of Kiel, Germany). The level of statistical significance was set at 95% (*p* < 0.05).

## 3. Results and Discussion

### 3.1. Morphological Analysis

**Figure 2:**
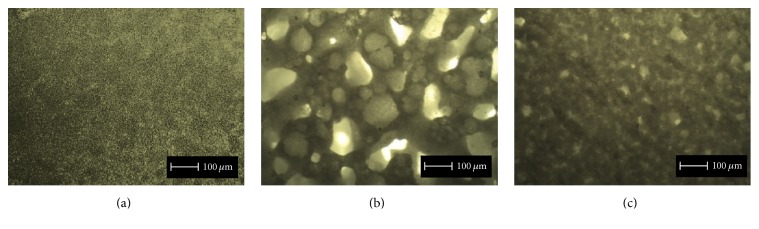
Micrographs (magnification: 40x) of PCL (a), PCL/CNF (b), and PCL/CNF/HANP (c) membranes.

Although the addition of CNF in the PCL polymeric matrix significantly influenced the surface morphology of the membranes, micrographs of the produced membranes confirm that the blends obtained by solvent casting were successfully produced, with homogeneous distribution of CNF within the polymer matrix (Figure 2). The adopted strategy of melting the PCL allowed an enhanced interaction between these two polymers.  Figure 2(b) clearly shows that the CNF incorporation promotes an increase in pore size. This outcome may be a consequence of the higher cellulose hydrophilicity, thus, potentially higher hygroscopy of the copolymer mixture than PCL alone, which could lead to external surface modification due to the contact with air moisture when the solution is deposited in the Petri dishes. This would lead to the surface energy increase with the addition of CNF, resulting in a pore size increment [26]. Additionally, the incorporation of HANP on the PCL/CNF composite promotes a pore size reduction when compared with PCL/CNF membranes (Figure 2(c)). This reduction was possibly due to HANP filling the observed empty spaces and/or promoting a higher cohesion of PCL and CNF in the solution used for material preparation.

**Figure 3:**
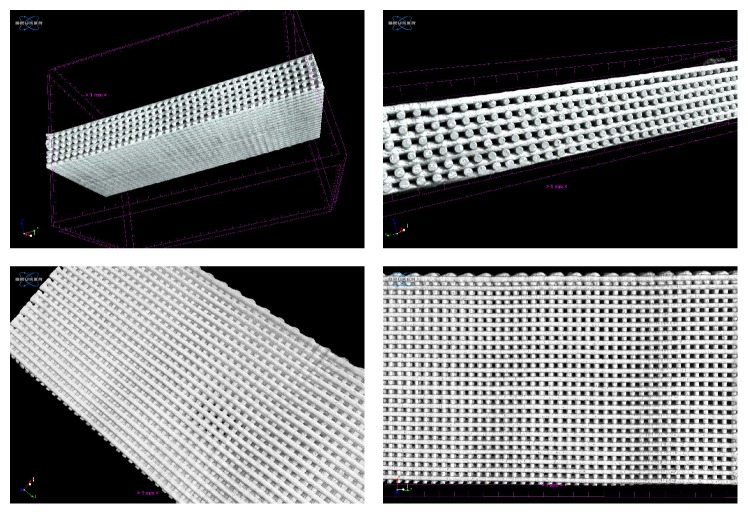
3D micro-CT images of PCL/CNF scaffolds.

The scaffolds were successfully produced presenting good geometric accuracy, fully interconnected channel networks, and highly controllable porosity. To verify the interconnectivity of the scaffolds and to calculate their porosity the micro-CT technique was used, as it enables the nondestructive visualization of the internal 3D structure of an object. All the obtained scans were similar, indicating that the proposed approach was precise, once the 3D structures exhibit good and accurate architecture and demonstrate the existence of connectivity between the pores (Figure 3). A high degree of interconnectivity is crucial to achieve a good viability of the inner parts of the scaffold, thus promoting a proper vascularisation of the graft and an effective tissue ingrowth* in vivo *[27].

The calculated porosities are presented in Table 1, showing that all the scaffolds revealed a valid porosity [24]. In fact, optimal balance should be aimed, as increasing porosity reduces the mechanical strength of the scaffold. Porosity is defined as the percentage of void space in a solid, with a morphological property being independent of the material [27]. Although the scaffolds present different compositions, their porosity percentage values were similar (*p* = 0.749), corroborating the geometric accuracy of the structures and the reliability of the extrusion equipment used for their production.

### 3.2. Surface Wettability of the Scaffolds

**Figure 4:**
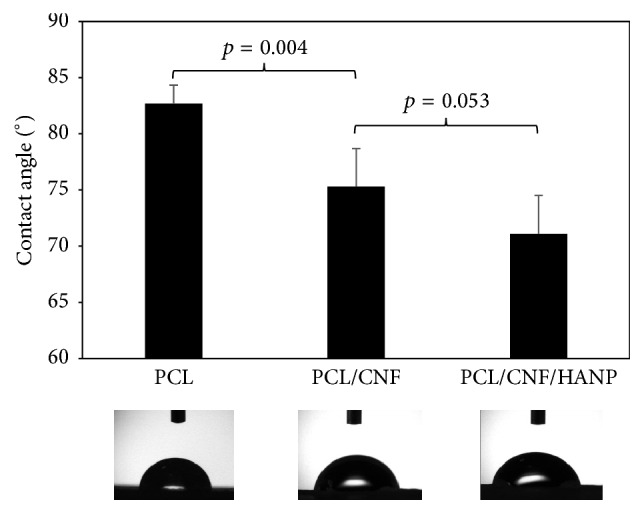
The static water contact angle of the produced nanocomposites.

The contact angle is a quantitative measure of the wetting of a solid by a liquid and is also dependent on the surface area, with higher surface energies being associated with lower contact angles [28]. The measured contact angles are presented in Figure 4 and it can be noticed that PCL surface had a higher (*p* < 0.0001) contact angle (82.7 ± 1.62°), corroborating a hydrophobic nature [29]. Furthermore, CNF are more hydrophilic than PCL; thus, as expected, the addition of CNF increased the surface wettability of the composite scaffolds and, consequently, the contact angle significantly decreased (*p* = 0.004) to 75.3 ± 3.39° [2]. Lastly, the hydrophobicity of the scaffolds was slightly reduced by the incorporation of HANP (71.1 ± 3.41°), without significant differences to the PCL/CNF composites (*p* = 0.053). These results clearly suggest that the addition of CNF on scaffolds may play a significant role in increasing its surface energies, which is beneficial for cell adhesion.

### 3.3. Thermal Analysis

**Figure 5:**
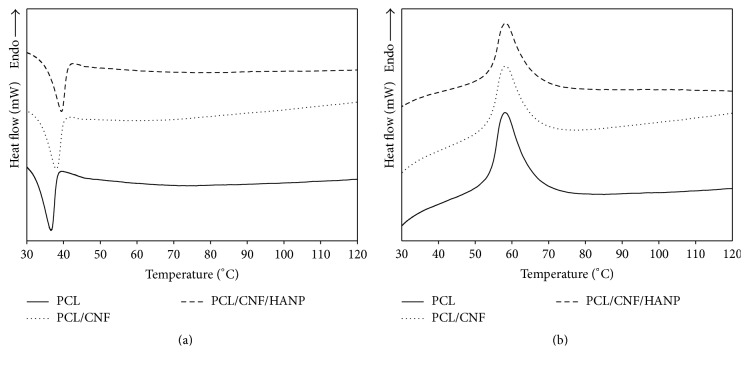
DSC curves of the processed samples: first cooling cycle (a) and second heating cycle (b).

**Figure 6:**
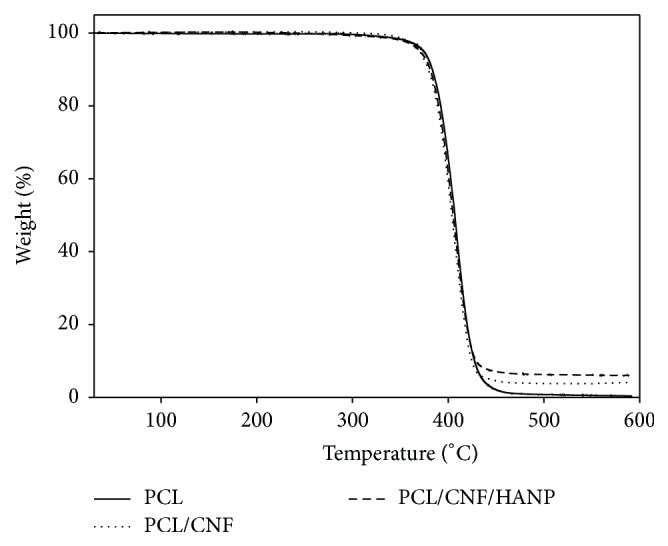
Thermogravimetric analysis of the produced nanocomposites scaffolds.

The thermal behaviour of the nanocomposites was studied by DSC and TGA (Figures 5 and 6) and the thermal parameters, including the melting temperature (*T*_*m*_), enthalpy of fusion (Δ*H*_*m*_), crystallization temperature (*T*_*c*_), degree of crystallinity (*X*_*c*_), decomposition temperature (*T*_*d*_), and mass loss, are summarized in Table 2 for all the samples. *X*_*c*_ was calculated using

(1) $$\begin{array}{c} X_{c}=\frac{\Delta H_{m}}{w\Delta H_{m}^{o}}, \end{array}$$

where Δ*H*_*m*_^*o*^ = 139.5 J/g is the enthalpy of fusion for 100% crystalline PCL [22] and *w* is the weight fraction of polymeric matrix in the composite.

In Figure 5(a) is presented the crystallization behaviour of the samples. *T*_*c*_ of the PCL/CNF composite slightly shifted to a higher temperature comparatively to pure PCL, suggesting that the crystallization of the PCL in the composite was affected by the addition of CNF. As a consequence, the crystallization started earlier in a nonisothermal process. On the other hand, the incorporation of CNF had no significant effect on *T*_*m*_ of PCL (Figure 5(b)), as supported by previous research [1, 2, 21]. Regarding the nanocomposites crystallinity, the *X*_*c*_ value of the PCL in the composite (PCL/CNF) decreased from 0.42 to 0.40 with the CNF addition. Therefore, CNF can have two different effects on PCL crystallization: (i) CNF may act as nucleating agents and promote PCL crystallization; or (ii) the polymer chain could be restricted by the incorporation of CNF, allowing a decrease in Δ*H*_*m*_ [2, 18, 21].

With the addition of HANP, the thermal behaviour of the nanocomposite did not change significantly. Comparative to PCL alone, results revealed a similar *T*_*m*_ and an increase of *T*_*c*_. At the same time, a lower heat of fusion (Δ*H*_*m*_) and nearly similar *X*_*c*_ were also observed. These results suggest that HANP can also change the crystallinity of the polymer and may accelerate the nucleation of the PCL segments [30–33].

The thermal stability of the scaffolds was investigated by thermogravimetric analyses, with a single weight loss step being observed for all the constructs (Figure 6). The decomposition temperature for PCL was consistent with values previously reported in literature [18, 34, 35]; however, the addition of CNF induced a significant decrease (Table 2). Furthermore, regarding mass loss of the nanocomposites, the presence of final residues at 600°C proved that PCL is almost completely degraded, while PCL/CNF and PCL/CNF/HANP composites have residual weights similar to CNF and HANP concentrations. To the best of our knowledge, this is the first study producing PCL scaffolds reinforced with CNF, with controlled architecture. Further research should focus on the behaviour adaptations made by different concentrations of CNF.

### 3.4. Mechanical Analysis

**Figure 7:**
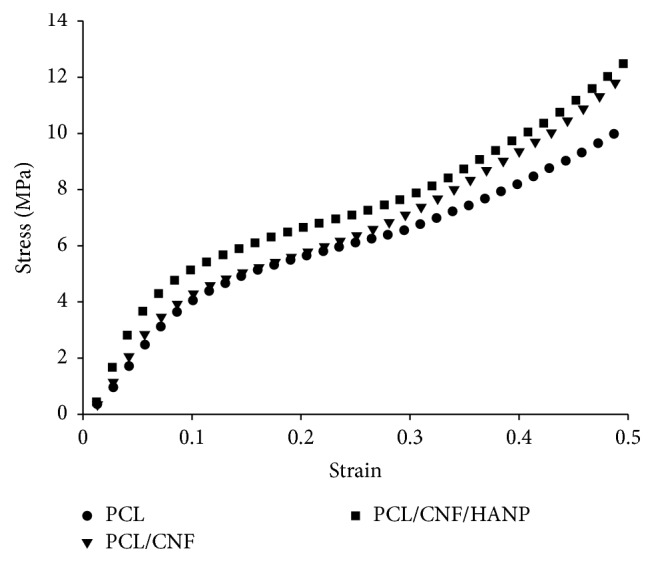
Stress-strain curve of the produced scaffolds.

The influence of CNF and HANP addition on the macromechanical performances of the scaffolds was investigated through compressive mechanical tests. The resultant compressive stress-strain curves, shown in Figure 7, demonstrate that the scaffolds presented the typical stress versus strain response of highly porous polymer scaffolds [12, 36]. The obtained curves are characterized by three different regions: a linear region at lower strain values, suggesting an initial rigid mechanical response, associated with elastic behaviour of the scaffolds; a region with lower stiffness; and, lastly, a region where a rise of stress with increasing strain is noticed, which is related to densification of the porous scaffolds [12, 35, 37].

The compressive modulus (*E*) and maximum stress (*σ*_max_) values obtained for all different scaffolds are presented in Table 3. The compressive strength of cortical and cancellous bone varies, depending on bone density, from 130 to 180 MPa and 5 to 50 MPa, respectively [38]. For the present study, the obtained values come near to the reported ones of the cancellous bone and significantly lower than the ones in cortical bone. Notwithstanding, the addition of CNF, even in a small amount, influenced the scaffolds mechanical response [21, 39], increasing the mechanical properties of the scaffolds (Table 3). Furthermore, the addition of the ceramic HANP improved the performance of the structures under compressive loads, which is in accordance with previous literature [34, 40, 41]. As previously mentioned, there is a lack of research on 3D structures of controlled architecture with CNF. However, this first study showed that the combination of CNF and HANP provided higher strength and rigidity to the constructed scaffolds.

### 3.5. Cytotoxicity Assessment

**Figure 8:**
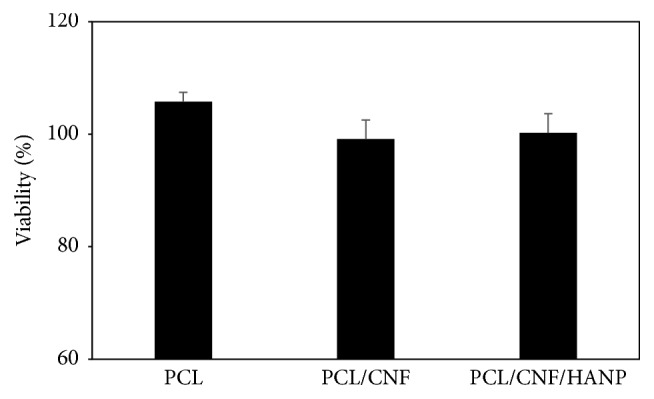
Cytotoxicity assessment: extract results.

One of the main features for scaffolds production is its biocompatibility. Scaffolds should aim to (i) be the responsible structures to work as substrate to adhesion, proliferation, and cell differentiation; (ii) establish a proper biomechanical environment for an organized tissue regeneration; (iii) allow the diffusion of nutrients and oxygen, and (iv) allow the encapsulation and release of cells and growth factors. As a first study using 3D scaffolds with controlled architecture, with the incorporation of CNF, it was mandatory to analyse their cell viability. The results of the extract assay (Figure 8) showed a viability of approximately 100% for all the conditions tested when compared to the control (105.8 ± 1.9 for PCL; 99.1 ± 3.4 for PCL/CNF; 100.2 ± 3.3 for PCL/CNF/HANP), without significant differences (*p* = 0.067) among constructs. Afterwards, a latex material was used to produce a positive control, showing high cell mortality when those are exposed to toxic lixiviates driven from this material, confirming sensibility of this cytotoxicity test to toxic materials.

**Figure 9:**
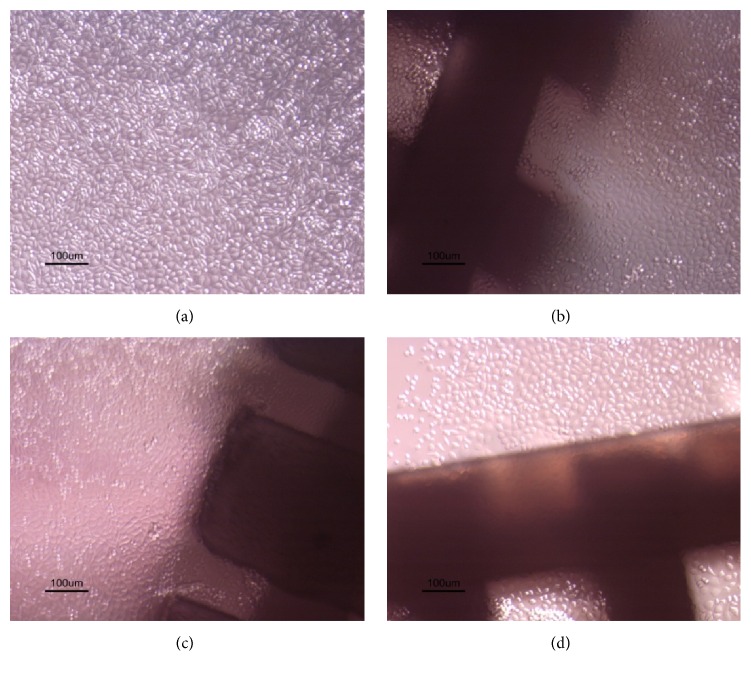
Cytotoxicity assessment: direct contact results of control (a); PCL scaffolds (b); PCL/CNF scaffolds (c); and PCL/CNF/HANP scaffolds (d).

Additionally, the contact direct results (Figure 9) corroborate the results obtained in the extract test, showing that cells in contact with scaffolds maintain their morphology and cell death at the interface with the materials was not observed. According to the results obtained it is possible to affirm that these nanocomposites turned out to be biocompatible.

## 4. Conclusions 

In this study poly(*ε*-caprolactone) membranes reinforced with cellulose nanofibers, with and without the addition of hydroxyapatite nanoparticles, were successfully produced by solvent casting. These membranes exhibit some differences in their morphology and the micrographs of the obtained composite reveal that the membranes had homogeneous distribution of CNF within the polymer matrix. The samples were subsequently processed by extrusion and the produced scaffolds present a fully interconnected network of internal channels and regular pore size, with similar porosity values and regular dimensions.

The incorporation of CNF and HANP into the PCL matrix had effects on surface wettability and thermal properties of the samples. The scaffolds energy surface increased with the addition of CNF and HANP. During cooling DSC scanning, the crystallization temperature of the nanocomposites started at higher temperatures than in the neat polymer. Mechanical compressive tests demonstrated the successful combination between PCL, CNF, and HANP. The mechanical properties of the PCL scaffolds were improved by incorporating CNF and further with HANP addition. The compressive and the elastic modulus of the composite scaffolds proved to be within the range of properties reported for human bone. The addition of CNF and HANP did not impair the biocompatibility of the obtained nanocomposites.

## Figures and Tables

**Table 1:** Mean ± sd values of the scaffolds porosity.

	PCL	PCL/CNF	PCL/CNF/HANP	*p*
Porosity (%)	49.0 ± 1.4	49.5 ± 2.1	50.5 ± 2.1	0.749

**Table 2:** Thermal properties of the produced scaffolds.

	PCL	PCL/CNF	PCL/CNF/HANP	*p*
*T*_*c*_ (°C)	36.6 ± 0.14	38.8 ± 0.30^a^	39.4 ± 0.07^a,b^	*<0.001*
*T*_*m*_ (°C)	58.0 ± 0.23	57.9 ± 0.21	58.2 ± 0.28	*0.158*
Δ*H*_*m*_ (j/g)	58.3 ± 2.01	54.6 ± 2.43	53.1 ± 0.79^a^	*0.019*
*X*_*c*_	0.42 ± 0.01	0.40 ± 0.02	0.41 ± 0.01	*0.107*
*T*_*d*_ (°C)	386.9 ± 1.75	381.0 ± 3.31^a^	383.4 ± 0.40	*0.038*
Mass loss (%)	98.3 ± 0.25	96.9 ± 0.83^a^	93.6 ± 0.43^a,b^	*<0.001*

^a^Significant difference from PCL; ^b^significant difference from PCL/CNF.

**Table 3:** Compressive mechanical properties of the scaffolds.

	PCL	PCL/CNF	PCL/CNF/HANP	*p*
Compressive modulus *E* (MPa)	54.42 ± 2.47	64.58 ± 5.94^a^	70.88 ± 8.60^a^	*0.004*
Maximum stress *σ*_max_ (MPa)	10.96 ± 0.92	11.35 ± 1.21	12.12 ± 0.82	*0.215*

^a^Significant difference from PCL.
